# A pilot study on the Spanish version of the Psychosocial Adjustment to Illness Scale (PAIS‐SR) with carers of people with Parkinson's disease

**DOI:** 10.1002/nop2.329

**Published:** 2019-07-01

**Authors:** Mari Carmen Portillo, Leire Ambrosio, Raquel Lanas Martín, Maria Victoria Navarta, MEugenia Ursua Sesma, Mario Riverol Fernández

**Affiliations:** ^1^ School of Health Sciences, NIHR Wessex CLAHRC University of Southampton Hampshire UK; ^2^ Department of Nursing Care for Adult Patients, School of Nursing University of Navarra Pamplona Spain; ^3^ Department of Psychiatry and Medical Psychology Clínica Universidad de Navarra Pamplona Spain; ^4^ San Juan Primary Health Centre Navarre Services of Health Osasunbidea Pamplona Spain; ^5^ Department of Neurology Clínica Universidad de Navarra Pamplona Spain

**Keywords:** carers, cross‐cultural adaptation, instruments, Parkinson's disease, pilot test, psychosocial adjustment to illness

## Abstract

**Aim:**

To report the cross‐cultural adaptation and pilot study of the ongoing validation of the Spanish version of the Psychosocial Adjustment to Illness Scale with carers of people with Parkinson's disease.

**Design:**

Cross‐cultural adaptation and pilot study with a cross‐sectional validation design of the Spanish version of the Psychosocial Adjustment to Illness Scale – Carers.

**Methods:**

Twenty‐one carers of people with Parkinson's disease from a Primary Care practice in Spain were recruited and completed the PAIS‐Carers, the SF‐36 Health Survey, the Brief COPE Inventory and an assessment form. SPSS 23.0 was used to determine viability/acceptability and preliminary aspects of internal consistency of the instrument.

**Results:**

Five of the seven domains presented floor effect (71.42%), and only one presented ceiling effect (14.28%). The internal consistency of the scale and domains showed acceptable values (over 0.7). The content validity of the Spanish version seemed satisfactory with positive comments in general from participants.

## 1 INTRODUCTION

Nowadays, the prevalence of chronic neurodegenerative conditions like Parkinson's disease (PD) has increased with the ageing of the population worldwide (Pringsheim, Jette, Frolkis, & Steeves, 2014) and it is estimated that around 10 million people have the condition around the world (European Parkinson's Disease Association, 2019).

PD involves changes for patients and families in all life spheres, and progressive adaptation becomes a key strategy for self‐management of the condition, normalization and family functioning (Ambrosio et al., 2015; Haahr, Østergaard, & Kirkevold, 2018; Kang & Ellis‐Hill, 2015; Mavandadi et al., 2014). Therefore, the psychosocial adjustment to PD is a complex process to several factors and mechanisms play a key role and, therefore, targeted interventions need to reflect the complexity and dynamism in clinical practice and integrate all the interactive components of the process and the multisystem approach where users, professionals and the community interact (Ambrosio et al., 2016, 2019; Derogatis, 1986; Derogatis & Derogatis, 1990; Roddis, Holloway, Bond, & Galvin, 2016; Wang et al., 2014).

## 2 BACKGROUND

Several studies have concluded that, although illness‐related symptoms or stress can influence the psychosocial adjustment to a long‐term condition (LTC) like PD, most of the times are factors related to family and social support, personality, expectations of illness management, financial status or coping skills that become paramount and transversal across conditions (Stanton, Revenson, & Tennen, 2007; Wang et al., 2014). Interestingly, these non‐illness‐related factors also gain importance for family carers and Navarta‐Sánchez et al. (2016) concluded that both people with PD and carers' quality of life is clearly influenced by the psychosocial adjustment to illness, which significantly depends on coping skills, regardless the disabling and degenerative nature of PD (Navarta‐Sanchez et al., 2017). Consequently, assessments and interventions should not only focus on the patient but also on the family, since it has been established that their experience of the adaptation process is comparable and that similar factors could influence this adjustment (Årestedt, Benzein, & Persson, 2015; Årestedt, Benzein, Persson, & Rämgård, 2016; Årestedt, Persson, & Benzein, 2014; Golics, Basra, Salek, & Finlay, 2013).

In this regard, the Psychosocial Adjustment to Illness Scale (PAIS‐SR) (Derogatis, 1986; Derogatis & Derogatis, 1990) evaluates the psychosocial adjustment process of a person with a health condition and/or its consequences and has versions for patients and carers. The scale is worldwide known and has been validated in several languages with people with different LTCs. Nevertheless, the scale has not been validated in a PD population and there is not a carer's version available in Spanish, despite the high relevance of this scale for carers of people living with PD and its implications for clinical use in Spanish‐speaking populations. This paper aims to present a brief report of the cross‐cultural adaptation and pilot study of the ongoing validation of the Spanish version of the PAIS‐SR with carers of people with PD.

## 3 THE STUDY

### 3.1 Design

We present results from a pilot study with a cross‐sectional observational design reporting the psychometric properties of the Spanish version (self‐report) of the PAIS‐SR with carers of people with PD.

### 3.2 Method

#### 3.2.1 Cross‐cultural adaptation process

After obtaining written permission from the author of the original PAIS‐SR, the translation of the English original version of the scale into Spanish was performed by a panel of four experts following the standard protocols used for transcultural adaptation of psychology questionnaires (Bonomi et al., 1996; Eremenco, 1998; Wild et al., 2005) (Table 1).

**Table 1:** Cross‐cultural adaptation of psychology questionnaires. Steps applied in this study

Steps	Process	Outcomes of the process
Step 1. Two forward translations from English (“ENGLISH 1”) into Spanish	Two independent translators	Translations “SPANISH 1” and “SPANISH 2”
Step 2. Reconciliation of the two Spanish versions.	Same independent translators compare the two versions (differences and wording refining)	Reconciled version “SPANISH 3”
Step 3. Back translation	Other bilingual translator with no previous contact translates to English	The resulting English version is “ENGLISH 2”
Step 4. Comparison of all versions in English and Spanish	All versions used previously (ENGLISH 1, SPANISH 2, SPANISH 3, and ENGLISH 2) were studied to find inaccuracies in the forward reconciled translation At this point, discrepancies individually found between the two versions were discussed	A more refined version (SPANISH 4)
Step 5. SPANISH 4 version of the scale reviewed	Spanish native expert naïve to the original version –but familiar to psychological scales–, reviewed the version to ensure natural wording Two of the translators previously involved also reviewed the Spanish 4 version for a natural wording	Minor changes to the previous version were made, obtaining version SPANISH 5
Step 6. Pilot study of SPANISH 5 version	Research study team	The SPANISH 5 version was tested in the pilot study as explained in this paper

#### 3.2.2 Setting, sampling and sample

The recruitment of participants took place in a Primary Health Centre of the northern region in Spain. Carers of people with PD living in the community and meeting the inclusion criteria in Table 2 were consecutively selected (Peduzzi, Concato, Kemper, Holford, & Feinstein, 1996; Stebbing, 2012).

**Table 2:** Inclusion criteria for family carers

Inclusion criteria	Description	Explanation/exceptions
Relationship with the PD patient	Participants will be family carers of a person diagnosed with PD at any stage	When more than one family member is involved in the person with PDs care, all will be invited to the study (family unit)
Permanent residence	Participants will live in Spain, be registered in the participating Primary health centre and have Spanish nationality	Essential for the cultural comprehension of the translation of the scale
Language	The participants' language will be Spanish or participants should be proficient enough in Spanish to complete the questionnaire	Essential for the cultural comprehension of the translation of the scale
Care at home	Participants will be caring for the person with PD at home	If the person with PD lives in a nursing home, participants will be the only person in charge of the patient's care
Other exclusion criteria	Unwillingness to participate, denied access	

#### 3.2.3 Data collection and instruments

The pilot study was completed in 2016, and the main validation study is under analysis. Data collection was planned as self‐reported. However, at all times, researchers were available to support participants should they have any question.

##### Carer's self‐report version of the PAIS‐SR

The scale has 46 items with Likert‐type answers grouped under a total of seven domains (Health Care Orientation, Vocational Environment, Domestic Environment, Sexual Relationships, Extended Family Relationships, Social Environment and Psychological Distress) (Derogatis, 1986; Derogatis & Derogatis, 1990; Rodrigue, Kanasky, Jackson, & Perri, 2000). Participants completed the SPANISH 5 version (Table 1) of the PAIS‐SR.

##### The 36‐item short form Health Survey (SF‐36)

The scale has 36 items with Likert‐type answers looking at positive and negative health aspects (Alonso, Prieto, & Anto, 1995; Vilagut et al., 2005) happening over the last 4 weeks in relation to physical functioning, role limitations due to physical health, role limitations due to emotional problems, energy/fatigue, emotional well‐being, social functioning, pain and general health.

##### Brief COPE scale (self‐report)

This is a multidimensional instrument with Likert‐type answers looking at different responses to stress (Carver, 1997). It contains 24 items under 12 subscales, which are self‐distraction, active coping, denial, substance use, use of emotional support, behavioural disengagement, venting, positive reframing, planning, humour, acceptance and religion.

##### Evaluation and sociodemographic forms

Apart from a sociodemographic form, participants completed an evaluation form to determine whether they had understood all the items, had found anything irrelevant or offensive and whether they had any comment or suggestion for additional questions to be included.

#### 3.2.4 Data analysis

Data did not follow a normal distribution, and the following non‐parametric statistics were applied to test the indicated attributes using SPSS 23.0. To determine the viability and acceptability of the cross‐culturally adapted scale, we analysed data quality registering missing data (accepting more than 95% of computable data). The limit for missing data was <5% in our study (Smith et al., 2005). Furthermore, the distribution of the punctuations with parameters like theoretical and observed range and descriptive statistics differences between median and mean were determined (arbitrary standard ≤ 10% maximum punctuation) (Martinez‐Martin et al., 2009). Floor and ceiling effect (<15%) and skewness were also tested (acceptable values: −1 and +1) (Hobart, Riazi, Lamping, Fitzpatrick, & Thomson, 2004).

Internal consistency was tested by Cronbach's alpha coefficient (criteria ≥ 0.70) (Scientific Advisory Committee of the Medical Outcomes Trust, 2002), domain‐total correlation (corrected for overlap; criterion value, *r*_s_ ≥ 0.30) (Martinez‐Martin et al., 2013) and inter‐item correlation (criterion value *r* ≥ 0.20 and ≤0.75) (Piedmont, 2014; Smith et al., 2005). Spearman rank correlation coefficient (rS) was used for testing these associations.

Utility and content validity. The percentage of responses completed, time taken for completion and perceptions of carers that participated in the pilot study were registered. Furthermore, the content validity was enhanced by following the cross‐cultural adaptation process of the scale where also experts from neurology and psychosocial adjustment to illness fields were involved.

#### 3.2.5 Ethical considerations

This study was approved by the Ethics Committee of the University of Navarra in Spain (reference 111/2013). Participants were accessed through the healthcare professionals in charge of their medical assistance in the centre under study. An informative letter and a consent form were provided.

## 4 RESULTS

Out of the 29 family carers invited to the study, 21 accepted to participate (see sociodemographic characteristics in Tables 3 and 4). A total of 85.7% of the participants were female and spouses of the person with PD. The mean age was 68.9 (*SD* 12.1) years (median: 72; range: 40–83 years). The mean time in which participants had been living with and caring for a person with PD was 4.1 (*SD* 3.3) years (median: 3; interquartile range: 1–12 years).

**Table 3:** Sociodemographic data of participants

Variable	Options	*N* = 21	%
Sex	Male	3	14.3
	Female	18	85.7
Marital status	Single	1	4.8
	Married/partner	19	90.5
	Separated/divorced	1	4.8
Working status	Full‐time job	1	4.8
	Housewife	8	38.1
	Unemployed	1	4.8
	Retired	10	47.6
	Other	1	4.8
Relationship with person with PD	Spouse	18	85.7
	Child	3	14.3
House adapted for care?	No	6	28.6%
	Adapted or partially adapted	15	72.4%
Maximum level of education	Can read and write	1	4.8%
	Primary	10	47.6%
	Sixth Form	5	23.8%
	University or equivalent	5	23.8%
Living area	Urban	20	95.2%
	Rural	1	4.8%
Income compared to country average	Lower	8	38.1%
	Similar	5	23.8%
	Higher	8	38.1%
Carer diagnosed of other conditions	Yes	13	61.9%
	No	8	38.1%

**Table 4:** Results from sociodemographic form and other measuring scales

	Age	Years as a carer	Total score brief cope	Total score SF‐36
*N*				
Complete	21	21	21	21
Missing	0	0	0	0
Mean	68.9	4.1	46.1	69
Median	72	3	48	65
*SD*	12	3.3	8.9	25.2
Skewness				
Theoretical range			24–96	0–100
Observed range	40–83	1–12	31–59	20–100
Percentiles				
25	65	1.5	37.5	50
50	72	3	48	65
15	77.5	5.5	52.5	92.5

There were no missing data, and data of items and domains were 100% computable. The mean scores in the scales were 46.1 (*SD* 8.9) for the Brief COPE; 69 (*SD* 25.2) for the SF‐36; and 32.7 (*SD* 17.1) for the PAIS‐Carers (Tables 4 and 5).

**Table 5:** Analysis results PAIS‐SR

	Total score PAIS‐SR	Domain 1 PAIS (healthcare orientation)	Domain 2 PAIS (vocational environment)	Domain 3 PAIS (domestic environment)	Domain 4 PAIS (sexual relationships)	Domain 5 PAIS (extended family relationships)	Domain 6 PAIS (social environment)	Domain 7 PAIS (psychological distress)
*N*								
Complete	21	21	21	21	21	21	21	21
Missing	0	0	0	0	0	0	0	0
Mean	32.7	9.2	2.7	3.1	5.1	1.6	5.9	5
Median	31	10	2.0	2	5	1	5	5
*SD*	17.1	4.3	2.3	3.6	3.7	1.9	5.5	2.7
Skewness	1.2	0.4	1.2	1.6	0.1	2.1	0.7	0.2
Theoretical range	0–138	0–24	0–18	0–24	0–18	0–15	0–18	0–21
Observed range	6–80	2–21	0–9	0–14	0–12	0–8	0–18	1–10
Percentiles								
25	23.5	6.5	1	0	1.5	0	1	5
50	31	10	2	2	5	1	5	2.5
15	38	11	4.5	4.5	8	2.5	11	7.5
Cronbach's alpha coefficient	0.86	0.80	0.75	0.77	0.80	0.78	0.75	0.77
Correlation domain‐total (corrected)	–	0.46	0.82	0.53	0.41	0.61	0.68	0.58
Correlation interdomain (range)	–	0.13–0.49	0.46–0.70	0.13–0.75	0.05–0.59	0.05–0.75	0.34–0.59	0.10–0.70

Looking further at the PAIS‐SR acceptability (Table 5), the difference between the mean and the median was lower than 10% in all domains and total score of the scale. No participant scored the maximum punctuation in the total score of the scale and in six of the seven domains.

Five of the seven domains presented floor effect (71.42%) and only one ceiling effect (14.28%). The total score and most domains of the PAIS‐SR showed acceptable values for skewness (except domain 5).

As shown in Table 5, Cronbach's alpha values were slightly over 0.7 for all domains and 0.8 for the total score of the PAIS‐SR, indicating acceptable internal consistency. The corrected domain‐total correlation showed values over 0.40 for all domains and interdomain correlations ranged from 0.10–0.75, being satisfactory for most domains.

The content validity was considered satisfactory as participants found the scale relevant mostly. Only three participants indicated that there were items whose comprehension was difficult, two participants stated that the scale was long and one participant found one item irrelevant. One comment referred to the inclusion of private life‐related domains in the scale (domestic environment and sexual relationships).

The pilot study resulted in some minor changes to the final Spanish version of the PAIS‐SR. The comments and suggestions reported in the pilot test were considered and discussed by the panel of three translators and an expert (Table 1) to make sure that the final version (Spanish 6) of the scale was ready for validation.

## 5 DISCUSSION

The purpose of this paper was to report results from the cross‐cultural adaptation and testing perspectives, and the results shown in this paper indicate that the adaptation of the original scale to Spanish language was adequately developed resulting in a viable scale ready for validation with a larger, more diverse and national population and sample.

From the pilot study results, we could initially conclude that the internal consistency, skewness, domain‐total and interdomain correlations of the scale were satisfactory. The interdomain correlation was acceptable indicating that the domains are clearly related to each other in the process of adjustment to PD from the carers' point of view. Previously, other reported and related scales have not shown this cohesion between domains in relation to living with an LTC (Ambrosio et al., 2016) when acceptance of the condition did not necessarily relate to the self‐management or the coping skills.

The sample size and diversity were limited because data were obtained from participants from only one health centre of a small locality in Spain, and most participants were female. Furthermore, it is important to highlight that PD is a neurodegenerative LTC which causes a great impact on the family carers. Therefore, this may have influenced some of the results presented here compare with the English version for carers of the PAIS‐SR validated with different populations of carers exposed to less burden or psychosocial impact (Greenwell, Gray, van Wersch, van Schaik, & Walker, 2015; Haahr et al., 2018; Kang & Ellis‐Hill, 2015).

Although the cross‐cultural adaptation process was developed rigorously according to international standards and the participants did not report any difficulty in understanding the Spanish version, this does not guarantee that the psychometric properties of the scale will be of high standards when applied to a population of carers of people with PD. They are important cultural factors which could have influenced the carers' perceptions and understanding of the Spanish version of the PAIS‐SR. Even in very well‐known and internationally applied scales like the PAIS‐SR, there are flaws and barriers for their application in clinical practice, especially when instruments are lengthy like the PAIS‐SR and include questions that entered domains of personal nature (Kolokotroni, Anagnostopoulos, & Missitzis, 2016; Perczek, Carver, & Price, 2000).

Time for reflection is needed as for its cost‐effectiveness and its use in practice nowadays. Finally, the PAIS‐SR could be considered more adequate for other type of LTCs with no degenerative progression, especially when it comes to overburdened carers. These aspects remained open, and at this stage, we need to wait for the results of the full validation study.

## 6 LIMITATIONS

This is the first study which has adapted the carers' version of the PAIS‐SR to Spanish with carers of people with PD. This is a pilot study taking place in a small geographical locality of Spain and does not represent the whole population of carers of people with PD. However, this is considered sufficient for a pilot testing and the main validation study is taking place at a national level, overcoming this limitation.

## CONFLICT OF INTEREST

No conflict of interest is declared.
